# Optimal dosing of angiotensin-converting enzyme inhibitors in patients with chronic heart failure: a cross-sectional study in Palestine

**DOI:** 10.4103/0256-4947.51794

**Published:** 2009

**Authors:** Waleed M. Sweileh, Ansam F. Sawalha, Tamara M. Rinno, Sa'ed H. Zyoud, Samah W. Al-Jabi

**Affiliations:** a College of Pharmacy, An-Najah National University, Nablus, Palestine; b Poison Control and Drug Information Center (PCDIC), An-Najah National University, Nablus, Palestine

## Abstract

**BACKGROUND AND OBJECTIVE:**

Because high-dose angiotensin-converting enzyme (ACE) inhibitor therapy is desirable in patients with chronic heart failure (CHF), we sought to determine the usage and dosing patterns of ACE inhibitors in CHF patients at a governmental hospital in Palestine.

**METHODS:**

This cross-sectional study was conducted between September 2006 and August 2007. All patients admitted with a confirmed diagnosis of CHF and an ejection fraction <40% were evaluated. After excluding patients with a caution/contraindication to ACE inhibitor use or not taking an ACE inhibitor, we determined the number of patients receiving optimal (captopril 150-300 mg/day, enalapril 20-40 mg/day, ramipril 5-10 mg/day) and suboptimal doses. We then conducted statistical analyses to evaluate associations between ACE inhibitor use and dosing and various demographic and clinical factors.

**RESULTS:**

Of the 165 patients initially evaluated, 69 (41.8%) had a caution/contraindication (n=28, 40.6%) or were not using an ACE inhibitor (n=41, 59.4%). Of the remaining 96 patients (70.1%), 49/96 (51%) were given an optimal dose while 47/96 (49%) were given a suboptimal dose. Of all patients with CHF and no contraindication (n=137), 88 (64.2%) were either receiving no ACE inhibitor or a suboptimal dose. Only the presence of hypertension was significantly associated with the use of an ACE inhibitor (*P*=.009, odds ratio=2.7). The use of an optimal dose was not significantly associated with any of the tested factors (age, gender, presence of hypertension, diabetes mellitus, renal dysfunction, ischemic heart disease or number of diagnosis).

**CONCLUSION:**

Underutilization and suboptimal dosing of ACE inhibitors was common. Since there is an abundance of evidence in favor of using high-dose ACE inhibitor therapy in patients with CHF, physicians need to be educated about proper dosing of these agents.

Chronic heart failure (CHF) is an increasingly common clinical condition with high morbidity and mortality.^1^ Current guidelines recommend that angiotensin-converting enzyme (ACE) inhibitors be used as first-line therapy for patients with CHF unless contraindicated.^2^ The doses given to patients with CHF should be those proven effective in randomized clinical trials.^3^–^6^ One study suggested that patients with CHF should be titrated to higher doses of ACE inhibitor therapy when possible.^7^ Another study also found that clinicians should attempt to reach high doses in patients with CHF.^8^ Even though low doses of ACE inhibitors have not been proven effective in reducing mortality,^9^ low doses are superior to no therapy.^7^ In light of this, we conducted this study to evaluate dosing with ACE inhibitors among patients with CHF at Al-Watani Hospital, a governmental hospital in Nablus, Palestine.

## METHODS

This cross-sectional study was conducted at the Al-Watani Hospital between September 2006 and August 2007. All patients admitted to the hospital during this period with a confirmed diagnosis of CHF and a maximum ejection fraction value (EF) of 40%, indicative of systolic heart failure, were initially evaluated for inclusion in the study. All information on the clinical condition of the patient and medication for CHF were obtained from the medical files and from the patient directly by a clinical pharmacist in the hospital. The study was approved by the hospital administration.

We excluded patients with cautions/precautions and contraindications to the use of ACE inhibitors, which include the following: pregnancy, history of angioedema, hypersensitivity to the drug, cough, hepatic dysfunction, hyperkalemia (> 5.5 mEq/mL), hypotension, renal insufficiency (creatinine clearance <30 mL/ min), and aortic or renal stenosis.^10^ For patients included in the study, the current type and dose of ACE inhibitor were determined. The use of an ACE inhibitor was defined as the current active use of the drug. The optimal dose was defined as any dose equal to or above the dose reported in the clinical trials that assessed mortality.^3^–^6^ For captopril, the optimal dose was considered any dose ≥150 mg daily; for enalapril, it was ≥20 mg daily, and for ramipril, it was ≥5 mg daily.^3^–^6^ Lower doses were considered suboptimal. If the patient was taking a suboptimal dose, but had recently been using the ACE inhibitor and was in the process of upward titration of the dose, he/she was considered to be taking an optimal dose.

Statistical analysis was carried out using statistical package for social sciences version 15. The chi-square test was used to test for significant associations between ACE inhibitor use and various categorical clinical and demographic factors as well as for association with optimal dosing. Results with *P*<.05 were considered statistically significant. The relative likelihood for the use of ACE inhibitor was estimated using an odds ratio (OR) calculated by means of cross tabulation analysis and provided with 95% confidence intervals (95% CI) for the odds ratios.

## RESULTS

During the study period, 165 patients with CHF and an EF <40%, indicative of systolic heart failure, were interviewed and their medical files were reviewed. Twenty-eight patients (17%) had cautions/contraindications to the use of ACE inhibitors and were excluded from the analysis. Reasons for exclusions included intolerance or allergy to ACE inhibitors (n=3; 10.7%), renal insufficiency (n=21; 75%), and hyperkalemia (n=4; 14.3%). One hundred thirty-seven patients (83%) met the inclusion criteria. The average age (standard deviation) of this study population was 68.6 (10.4) years and 73 (53.3%) were females. Of the patients included, 95 (69.3%) had hypertension, 72 (52.5%) had diabetes mellitus, and 39 (28.4%) had a creatinine clearance <60 mL/ minute (Table 1).

**Table 1:** Characteristics of 137 chronic heart failure patients based on the use of an ACE inhibitor.

Factor		Number of patients using ACE inhibitor (n=96)	Number of patients not using ACE inhibitor (n=41)	Odds ratio vs. other category (95% confidence intervals for use of ACE inhibitor)	*P* value
Sex	Male	45 (46.9%)	19 (46.3%)	1.02 (0.5-2.1)	.95
	Female*	51 (53.1%)	22 (53.7%)		

Hypertension	Yes	73 (76%)	22 (53.7%)	2.7 (1.3-5.9)	.009
	No*	23 (24%)	19 (46.3%)		

Ischemic heart disease	Yes	34 (35.4%)	12 (29.3%)	1.3 (0.6-2.9)	.50
	No*	62 (64.6%)	29 (70.7%)		

Diabetes mellitus	Yes	55 (57.3%)	17 (41.5%)	1.9 (0.9-3.9)	.09
	No*	41 (42.7%)	24 (58.5%)		

CrCl (mL/min)	≥60	68 (70.8%)	30 (73.2%)	0.9 (0.4-2.1)	.78
	<60*	28 (29.2%)	11 (26.8%)		

Age (years)	< 65	35 (36.5%)	12 (29.3%)	1.4 (0.6-3)	.42
	≥ 65*	61 (63.5%)	29 (70.7%)		

CrCl: creatinine clearance.

* Reference category.

**Figure 1:**
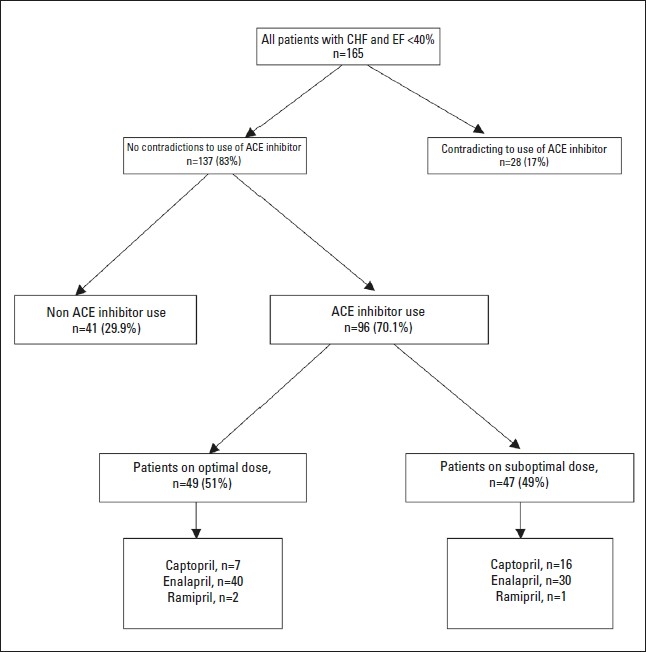
Overview of study results.

Of the patients included in the analysis, 96 (70.1%) were using an ACE inhibitor while 41 (29.9%) were not (Figure 1). The use of an ACE inhibitor was significantly associated with hypertension, but not with sex, age, diabetes, ischemic heart disease, or renal function.

However, diabetic patients and patients younger than 65 years old were more likely to use an ACE inhibitor (Table 1). The types of ACE inhibitors and dosing information are shown in Table 2.

**Table 2:** Summary of the types and doses of ACE inhibitors used in patients with CHF.

	Captopril	Enalapril	Ramipril
Patients, n (%)	23 (17%)	70 (51%)	3 (2%)
Patients taking optimal dose, n (%)	7 (30%)	40 (57%)	2 (67%)
Patients on sub-optimal dose, n (%)	16 (70%)	30 (43%)	1 (33%)
Optimal dose (range in mg)	150-300	20-40	5-10
Mean (SD) daily dose (mg/d)	34 (15.5)	15.5 (11.9)	2.9 (1.9)
Median dose received (mg)	25	10	2.5
Minimum dose used (mg/d)	12.5	5	1.25
Maximum dose used (mg/d)	75	50	5

Of the patients using an ACE inhibitor, 49/96 (51%) were taking optimal doses while 47/96 (49%) were taking a suboptimal dose. Those taking suboptimal maintenance doses had no identifiable contra-indication to the optimal dose. A total of 88/137 (64.2%) patients with CHF were either not taking an ACE inhibitor or were using a suboptimal dose in the absence of a contraindication to increase the dose to the optimal dose. Of the two most commonly used ACE inhibitors, dosage was significantly more optimal with enalapril compared to captopril (57.1% versus 30.4%; *P*=.026).

Statistical analysis indicated that none of the tested factors (age, gender, presence of hypertension, diabetes mellitus, renal dysfunction, ischemic heart disease or number of diagnosis) were significantly associated with use of an optimal dose of an ACE inhibitor, and thus no valid model could be built to predict the use of an optimal dose. However, patients <65 years of age (odds ratio=2.3, 95% CI 1 - 5.4), or patients with creatinine clearance >60 mL/min (odds ratio=2.0, 95% CI 0.8-4.8), or diabetic patients (odds ratio=1.8, 95% CI 0.8-4) were more likely to be using optimal doses.

## DISCUSSION

Chronic heart failure is one of the major causes of mortality worldwide. Furthermore, the economic consequences of its management constitute a true burden on the health system,^11^ which is why optimization of CHF therapy is of great importance. During the past 20 years, the many trials conducted in patients with CHF have concluded that ACE inhibitor use confers a 16% to 20% reduction in mortality.^12^

Underutilization or use of a suboptimal dose of an ACE inhibitor in patients with CHF seems common.^13^ Variables that could affect the use of optimal doses of an ACE inhibitor include both physician and patient-dependent factors. One study found that regardless of physician specialty, approximately one-third of ACE inhibitor prescriptions are for suboptimal doses. Interestingly, the study also showed that general practitioners tend to prescribe higher doses than specialists.^14^ Another study, which examined dosing and compliance with ACE inhibitors, concluded that efforts aimed at enhancing patient compliance and prescribing adequate dosages are needed.^15^ Another study that quantified the extent and determinants of underutilization of ACE inhibitors for patients with CHF found that family practitioners and general internists probably underutilize ACE inhibitors more often compared with cardiologists.^16^ In the current study, the lack of association between ACE inhibitor use and most of the tested factors might suggest that ACE inhibitor use is determined by individual factors and that there is no particular pattern of use.

In our study, dosing was more optimal for patients who took enalapril. One possible explanation for more suboptimal dosing with captopril is that patients using that agent need to take three tablets daily to achieve the optimal target range because the highest available strength of captopril is 50 mg. In contrast, patients using enalapril need to receive only once or twice daily dosing of 10 mg or 20 mg of enalapril to reach the optimal target range.

Our study had some limitations. First, it is possible that the percentages of patients using optimal doses were underestimated. For example, some patients may have tried higher doses in the past, but had to discontinue because of an inability to tolerate the higher dose. Second, patients enrolled in the study are those who have governmental medical insurance. Therefore, the finding in this study could not be extrapolated to clinical practice in private clinics. To improve the dosing of ACE inhibitors in patients with CHF, continuing medical education for physicians is highly recommended. Furthermore, clinical pharmacists are needed to review medications and give advice to practitioners in hospitals.
